# Medical Meeting

**Published:** 1852-09

**Authors:** J. E. Roberts, S. Y. Baldwin

**Affiliations:** President; Secretary


*Medical Meeting.*

At a Meeting of the Medical Profession of Macon co., held at the Court House in Decatur, July 17, 1852, pursuant to previous notice; J. E. Roberts, M. D., was chosen Chairman, and S. Y. Baldwin, M. D., Secretary. Whereupon, a committee of three, consisting of Drs. Kellar, Trowbridge, and Baldwin, reported the following premeable and constitution which was unanimously adopted:

Whereas, it has been shown by experience that the association of persons engaged in the same pursuit, facilitates the attainment of their common objects, therefore,

Art. 1—Resolved, That it is expedient for the regular practitioners of the profession of medicine of the county of Macon, to form themselves into an association to be called the Medical Society of Macon county, for the protection of their interests, for the maintainance of their honor and respectability, for the advancement of their knowledge, and the extension of their usefulness.

Art. 2. That, for the furtherance of the above object, and for the better organization, the officers of such society shall consist of a president, vice-president, secretary, and treasurer, to be chosen by a majority of the members present and to hold their office for the term of one year.

Art. 3. That the president, or in his absence, the vice president, shall preside at all meetings of the society, shall decide all questions of order that may arise, according to parliamentary usage, and in the event of a tie, shall have a casting vote. That the secretary shall keep a faithful minute and record of the proceedings of said society, and shall have the power, by and with the concurrence of the president, to call a special meeting thereof. That the treasurer shall receive all funds paid into said society, and disburse the same according to the wishes of said society, as expressed at any regular meeting and make report thereof at the end of the year.

Art. 4. That any regular practitioner in good standing, may become a member of this society, by subscribing to this constitution, by a vote of two-thirds of the members present at any regular meeting.

Art. 5. The regular meetings of this society shall be held on the first Saturday of every month, for the discussion of such subjects as may be chosen by the society, and for the transaction of such other business as may properly come before the society.

Art. 6. Those members of the profession present at the formation of this society, shall be and are hereby constituted members thereof.

Art. 7. This society shall be governed by the code of medical ethics, adopted by the Illinois State Medical Society.

Art. 8. This constitution may be altered or amended by a vote of two-thirds of the members present, at any regular meeting of the society.

The society as above organized, proceeded to the election of officers. Whereupon Joseph King, M. D., was chosen President,

S. T. TROWBRIDGE, M. D., Vice-President, A. L. KELLAR, M. D., Treasurer, S. Y. BALDWIN, M. D., Secretary.

On motion, the Secretary was instructed to furnish a copy of these proceedings for publication in the *North-Western Medical Journal* and *Shoaff's Family Gazette.*

On motion, the society adjourned to meet the first Saturday in August next, at 2 o'clock P. M.

J. E. ROBERTS, M. D., President.
S. Y. BALDWIN, M. D., Secretary.

